# Recent Trends in Bio-nanomaterials and Non-invasive Combinatorial Approaches of Photothermal Therapy against Cancer

**DOI:** 10.7150/ntno.91356

**Published:** 2024-02-17

**Authors:** Nitisha Beniwal, Anamika Verma, Chandra Lekha Putta, Aravind Kumar Rengan

**Affiliations:** Department of Biomedical Engineering, Indian Institute of Technology Hyderabad, Kandi, Sangareddy, Telangana-502285, India.

**Keywords:** cancer, bio-nanomaterials, photothermal therapy, combination therapy, photothermal agents, theranostics

## Abstract

In 2020, approximately 10 million deaths worldwide were attributed to cancer, making it the primary cause of death globally. Photothermal therapy (PTT) is one of the novel ways to treat and abolish cancer. PTT significantly impacts cancer theranostics compared to other therapies like surgery, chemotherapy, and radiotherapy due to its remarkable binding capability to tumor sites and lower invasiveness into normal healthy tissues. PTT relies on photothermal agents (PTAs), which generate heat by absorbing the near-infrared (NIR) light and destroying cancer cells. Several PTT agents remain longer in the reticuloendothelial system (RES) and induce toxicity, restricting their use in the biomedical field. To overcome this problem, the usage of biodegradable nano-photothermal agents is required. This review has discussed the PTT mechanism of action and different types of novel bio-nanomaterials used for PTT. We also focussed on the combinatorial effects of PTT with other cancer therapies and their effect on human health. The role of LED lights and mild hypothermia in PTT has been discussed briefly in this review.

## 1. Introduction

Cancer treatment is the most prominent challenge faced by the healthcare sector today 1. Due to high mortality and spreading rate, it is a life-threatening disease to humans worldwide, causing almost 10 million deaths in 2020, nearly one death for six people, predicting 19.3 million new cases globally 2. For Centuries, scientists have put their effort into searching for a successful cancer therapy. Currently, the treatments used for cancer are chemotherapy, radiotherapy, and surgery, with not-so-effective results accompanied by grievous side effects. However, many more technologies have developed with time, like photodynamic therapy (PDT), gene therapy, immunotherapy, and photothermal therapy (PTT), which are advanced and have become more popular because of their characteristics 3. PTT gained much attention in these treatment modalities because of its higher specificity, lower side effects, strong anti-cancer ability, and non-invasiveness 4. PTT uses photothermal agents (PTAs) that generate a photothermal impact by absorbing light energy and converting it into heat, raising the tumor cell's temperature, which induces cell death because cancer cells are less tolerant to heat 3. PTAs can be constructed on a nano-scale by modifying various mechanisms to achieve active and passive targeting of tumor sites, which causes the superimposed targeting effect of PTT. Secondly, the laser is only exposed to the cancer cell, so toxicity to normal cells is reduced. Hence, the critical aim of cancer treatment is to abolish tumor cells without affecting healthy tissues 5,6. Over the decade, various organic/inorganic nano-compounds such as metal-based nanomaterials (Gold (Au), Silver (Ag), platinum (Pt)), NIR dyes (indocyanine green, IR780, IR825, cypate), carbon-based nanomaterials (graphene and carbon nanotubes), Conducting polymers (polypyrrole and polyaniline), bio-degradable proteins/peptides, melanin like polymers are used as PTAs. Despite all these, PTAs exhibiting NIR absorbance are yet to study their full potential *in vivo* because of their non-biodegradability, poor pharmacokinetics, lower immunogenicity, higher toxicity, and inflammatory side effects. Hence, PTAs should generate a high clinical translation effect and maximize the therapeutic potential of PTT 7. To achieve this, bio-degradable nanomaterials like conducting polymers, melanin-like polymers, bio-degradable proteins/peptides, carbon-based nanomaterials, and small molecular organic dyes are synthesized because of their inborn biocompatibility/biodegradability, recognized degradation mechanism and established metabolic pathways.

The United States Food and Drug Administration (FDA) reported that PTAs in clinical translation must be eliminated from the human body in a given period. Mechanisms that can elicit the degradation of nanomaterials by inducing stimulation have a superior advantage 4. Although PTT has a remarkable effect in clinical therapeutics, it also has limitations, especially in treating tumors on edge; light penetration to the deep tumors is inadequate, and these cells were removed incompletely. This can also cause the regrowth and spread of tumors to other body parts. Nowadays, monotherapy involving PTT is not strong enough to cure cancer entirely due to the limitations mentioned above. To improve the overall efficacy of PTT, many synergistic approaches like PDT (photodynamic therapy), immunotherapy, gene therapy, and chemotherapy are being harnessed to achieve exact diagnosis and accuracy of tumor treatment altogether, providing an excellent strategy to overcome many cancers and other ailments 8. These combinatorial approaches are practical over monotherapy in two apparent ways, i.e., reducing the risk of heat generation causing skin burning, scarring, and damage of tissues near tumor cells and minimizing the tumor metastasis and inflammatory side effects 9.

Nanozymes, emerging as a cutting-edge class of synthetic enzymes, present promising prospects in the realms of biomedical engineering and the treatment of diseases. The optimization of nanozymes through the integration and harmonization of their diverse intrinsic functionalities holds significant potential for enhancing their efficacy within biological systems.

Fan et al., developed a groundbreaking nanozyme with a distinctive yolk-shell structure by integrating a solitary gold nanoparticle core with porous hollow carbon shell nanospheres (Au@HCNs). This innovative nanostructure, Au@HCNs, manifests enzyme-like functionalities akin to horseradish peroxidase and oxidase when exposed to an acidic milieu, showcasing its proficiency in generating reactive oxygen species (ROS). Notably, the ROS generation by Au@HCNs is markedly heightened upon exposure to 808-nm light irradiation, capitalizing on the photothermal effect commonly employed in tumor therapy. In-depth investigations, encompassing both cellular and animal studies, underscore that the amalgamation of light-enhanced ROS and photothermal therapy leads to remarkably effective tumor eradication. These findings suggest a synergistic potential wherein the inherent enzyme-like activity and photothermal conversion of nanozymes can be harnessed for a pioneering approach to tumor treatment. This study serves as a compelling proof-of-concept for catalytic-photothermal therapy targeting tumors through the utilization of nanozymes 10.

Yang et al., has been successfully developed a highly efficient platinum (Pt)-carbon-integrated nanozyme with advantageous catalase-like activity and photosensitizing properties by incorporating an ultrasmall Pt nanozyme into a carbon nanozyme derived from metal-organic framework (MOF) through an in situ reduction strategy. The integration of the Pt nanozyme markedly enhances the catalase activity of the carbon nanozyme, enabling effective catalysis of the decomposition of endogenous hydrogen peroxide to generate oxygen and thereby improving the efficacy of photodynamic therapy. Furthermore, the incorporation of the Pt nanozyme augments the intrinsic photothermal performance of the carbon nanozyme. The synergistic effects of enhanced catalase-like activity and improved photothermal properties contribute to the Pt-carbon nanozyme's exceptional ability to inhibit tumor growth in vivo 11.

Nie et al. developed the hemostatic matrix system Surgiflo@PCN, incorporating Surgiflo, a versatile multispace structure designed for effective penetration into diverse tumor cavities to mitigate postoperative hemorrhage. The system features porous palladium-copper nanoclusters (PCNs) with tunable enzyme-like activities (oxidase, peroxidase, and catalase) that induce the generation of reactive oxygen species (ROS) upon near-infrared (808 nm) laser irradiation. Upon introduction into the resected tumor cavity, Surgiflo@PCN initiates a dual-action process. Firstly, it directly eliminates glioma cells through ROS generation and photothermal therapy (PTT). Subsequently, it induces immunogenic cell death by enhancing oxidative stress and PTT with PCN, thereby reversing the immunosuppressive tumor microenvironment and promoting an enhanced antitumor immune response 12.

Zhang et al. have pioneered the development of single-atom catalysts (SACs), wherein metal active sites are meticulously isolated on a supportive substrate and stabilized through coordination with atoms like oxygen, nitrogen, sulfur, and others. This innovative approach maximizes the utilization efficiency of metal atoms. Thanks to advancements in synthetic strategies, characterization techniques, and computational models, numerous SACs exhibiting remarkable catalytic performance across a spectrum of reactions have been successfully engineered. The optimization and enhancement of catalytic selectivity and activity are pivotal considerations within the realms of nanotechnology and biomedicine 13.

Zhang et al. have developed an internally implantable biodegradable hydrogel and an extracutaneously applicable antioxidant bioadhesive to concurrently address postoperative tumor recurrence and mitigate radioactive skin injury following adjuvant radiotherapy. The biodegradable silk fibroin/perfluorocarbon hydrogel, loaded with doxorubicin (DOX), is created through consecutive ultrasonication-induced β-sheets-crosslinked amphiphilic silk fibroin/perfluorocarbon/DOX nanoemulsion. This innovative hydrogel facilitates continuous oxygen release in a physiological environment, enhancing hypoxia and radiotherapy sensitivity. Simultaneously, DOX is gradually released, ensuring an effective anti-cancer outcome. A stretchable bioadhesive is crafted through the copolymerization of α-thioctic acid and N, N-diacryloyl-L-lysine, with gold nanorods and gallic acid incorporated for gentle photothermal therapy and antioxidant capabilities. Controlled release of gallic acid and mild photothermal therapy, triggered by near-infrared light, efficiently neutralize excess free radicals generated by radiotherapy, promoting effective radioactive wound healing. In vivo animal studies affirm the efficacy of this methodology, demonstrating that post-tumor resection administration of the hydrogel, coupled with the concurrent application of an antioxidant bioadhesive patch, effectively inhibits tumor recurrence and alleviates the progression of skin radiation damage 14. In this review, we have epitomized the recent advancements in bio-nanomaterials used for PTT and discussed the combinatorial effect of PTT with other synergistic therapies. The role of LED lights and mild hypothermia in PTT has been discussed briefly in this review.

## 2. Mechanism of PTT Action

**Figure 1:**
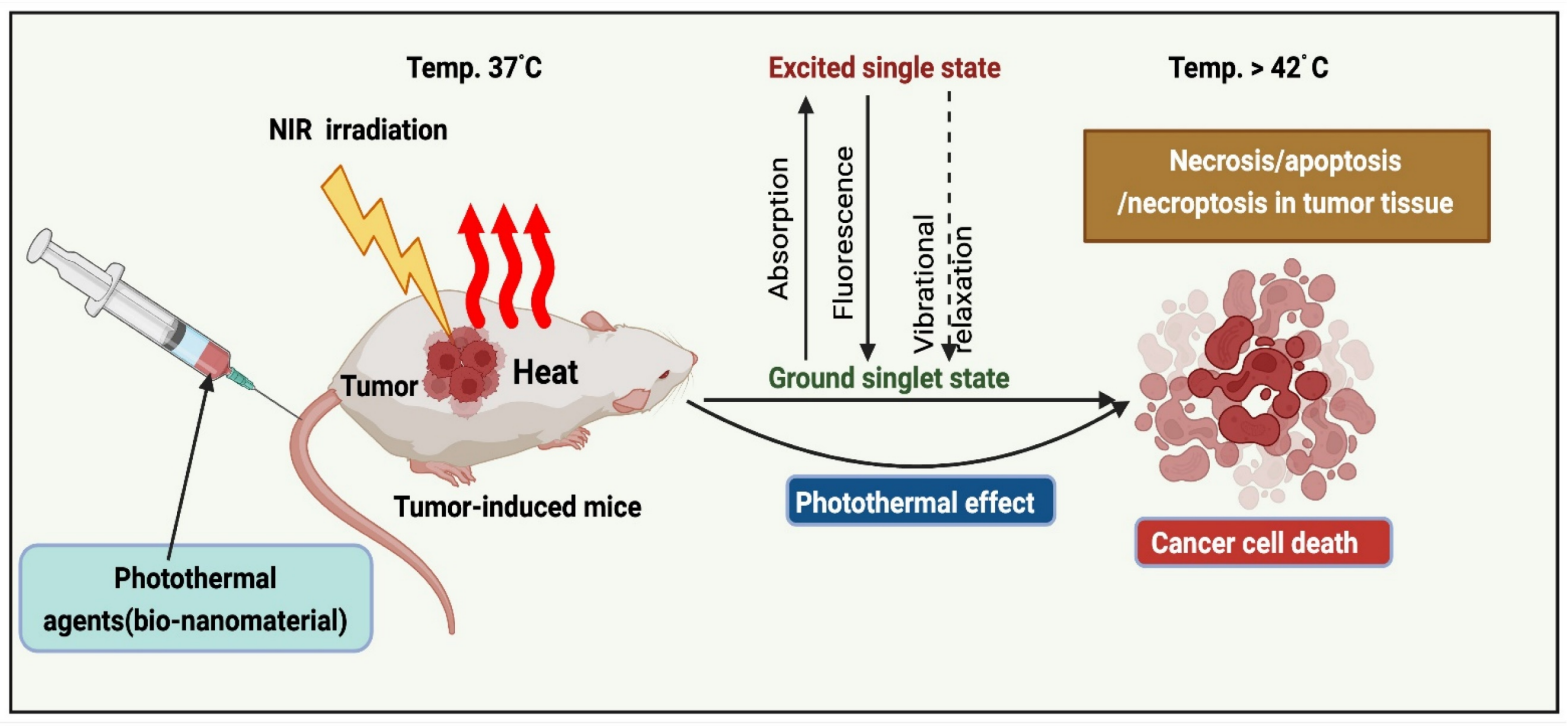
Mechanism of PTT action.

The implementation of PTT to remove solid tumors has grown exponentially over the past decade 15. PTAs at the tumor sites (primary tumors and early local metastases) were exposed to near-infrared (NIR) light. Transfer of energy by electron-electron relaxation of photothermal agents increases the temperature, leading to hyperthermia and cancer cell death at target sites (Figure 1) 16. Photothermal agents are employed across various wavelength ranges, including NIR-I (700-950 nm), NIR-II (1000-1350 nm), NIR-III (1600-1870 nm), and NIR-IV (2100-2300 nm). As irradiation sources, current PTT research focuses primarily on lasers in the NIR-I window. In addition, NIR-II, precisely the NIR-IIa window (1300-1400 nm), is believed to be more effective for diagnostic purposes and therapy due to its superior infiltration and excellent permissible exposure compared to the NIR-I window 17.

Three fundamental mechanisms of cell death are triggered by PTT: 1) Cell necrosis, 2) Apoptosis, and 3) Necroptosis.

**Figure 2:**
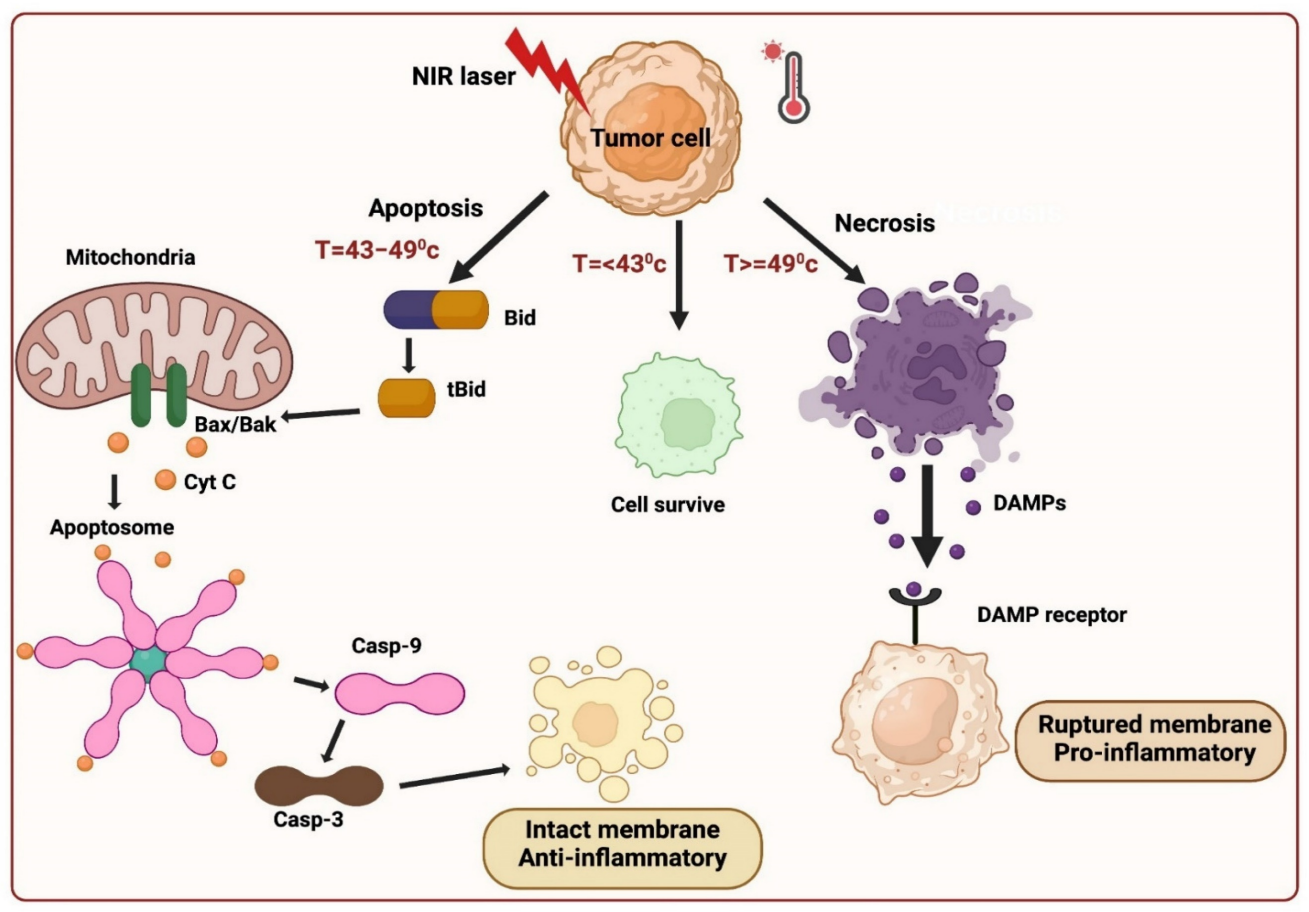
Cell death mediated by PTT.

Cell necrosis (NE) represents an unplanned cellular demise characterized by an enlargement of the cytoplasm, severe organelles damage, and the plasma membrane's eventual rupture 13. This process releases intracellular contents, including damage-associated molecular patterns (DAMPs), into the surrounding extracellular environment. This aberrant release can provoke harmful inflammatory and immunogenic responses, rendering necrosis an unfavorable pathway for cell death 10. In contrast to necrosis, apoptosis maintains the integrity of the cell membrane and sends "eat me" signals, such as phosphatidylserine (PS), which relocated to the membrane's outer surface, serving as markers for phagocytosis. When encountered by phagocytes, apoptotic cells undergo transformations that discourage inflammation, yielding a distinct and more desirable outcome compared to the inflammatory consequences associated with necrosis. Inducing necrosis through physical disruption of the cell membrane offers the advantage of causing immediate cell death, and it is not prone to resistance that can affect other therapeutic approaches. The initiation of apoptosis in cells is contingent upon the initial temperature and is primarily influenced by two key factors: laser power and exposure duration. When the irradiation conditions are optimal, PTT can trigger apoptosis instead of necrosis. Extensive research has demonstrated that, particularly under low-energy irradiation, the intrinsic pathway is predominant in facilitating PTT-induced apoptosis. Whether the extrinsic or intrinsic mitochondrial pathway is the primary route to cell death in apoptosis is a fascinating topic to explore. Analyzing the molecular signaling pathways involved in the cellular response to PTT can provide insights into this matter. The extrinsic pathway, triggered when specific ligands bind to "death receptors" on the cell surface, is unlikely to be the primary mode of cell death induced by PTT. On the other hand, the intrinsic pathway is activated in response to cellular stress, such as DNA damage and heat shock, making it a more probable mediator of the cell's response to PTT. In the intrinsic pathway, cell stress activates molecules like Bak and Bax, which subsequently initiate mitochondrial outer membrane permeabilization and cytochrome c release into the cytoplasm. Cytochrome c then interacts with Apaf-1, deoxyadenosine triphosphate (dATP), and procaspase 9 to form a complex known as the apoptosome. This apoptosome, in turn, activates caspase-9, which cleaves and activates caspase-3, setting off a cascade of events downstream that ultimately leads to the cell's demise and its subsequent phagocytosis demonstrated in (Figure 2).

It is important to note that there is a degree of communication between the extrinsic and intrinsic apoptosis pathways. When death receptors are engaged by their respective ligands, caspase-8 is activated and plays a role in cleaving Bid into tBid. tBid then moves to the mitochondria, where it activates Bax/Bak and initiates the release of cytochrome c. This illustrates the intricate interplay between these pathways 15.

In PTT, when cancer cells are exposed to high temperatures (above 42 °C), they typically die via necrotic or apoptotic mechanisms. Moreover, researchers have identified a novel mechanism in PTT for cancer cell death referred to as necroptosis, which is identical to a passive, unregulated necrotic cell death pathway and a highly regulated cell death progression 18. In this mechanism, gene expression configurations were altered at a temperature of 41 °C, releasing heat-shock proteins, which mitigated thermal damage to cancer cells. The heat generated from NIR light irradiation causes irreversible tissue damage. When the temperature reaches 46 °C, cancer cells experience necroptosis and apoptosis, while temperatures above 49 °C lead to cancer cell necrosis. In the case of necrosis, the elevated heat disrupts the membrane of cancer cells, resulting in the release of cytoplasmic components and triggering an inflammatory response. Cell death occurs at 46 °C due to ischemia and microvascular thrombosis, resulting in protein denaturation and cell membrane destruction. The apoptotic pathway is highly regulated and does not induce inflammation, which makes it an appropriate method for abolishing cancer cells. PTT widely identified this distinction between cancer treatment cell-death mechanisms. Recent studies have revealed that necroptosis, a form of programmed cell death, can enhance the effectiveness of antitumor therapies and overcome drug resistance in tumor cells by disrupting apoptosis 14.

## 3. Bio-nanomaterials used for PTT

**Figure 3:**
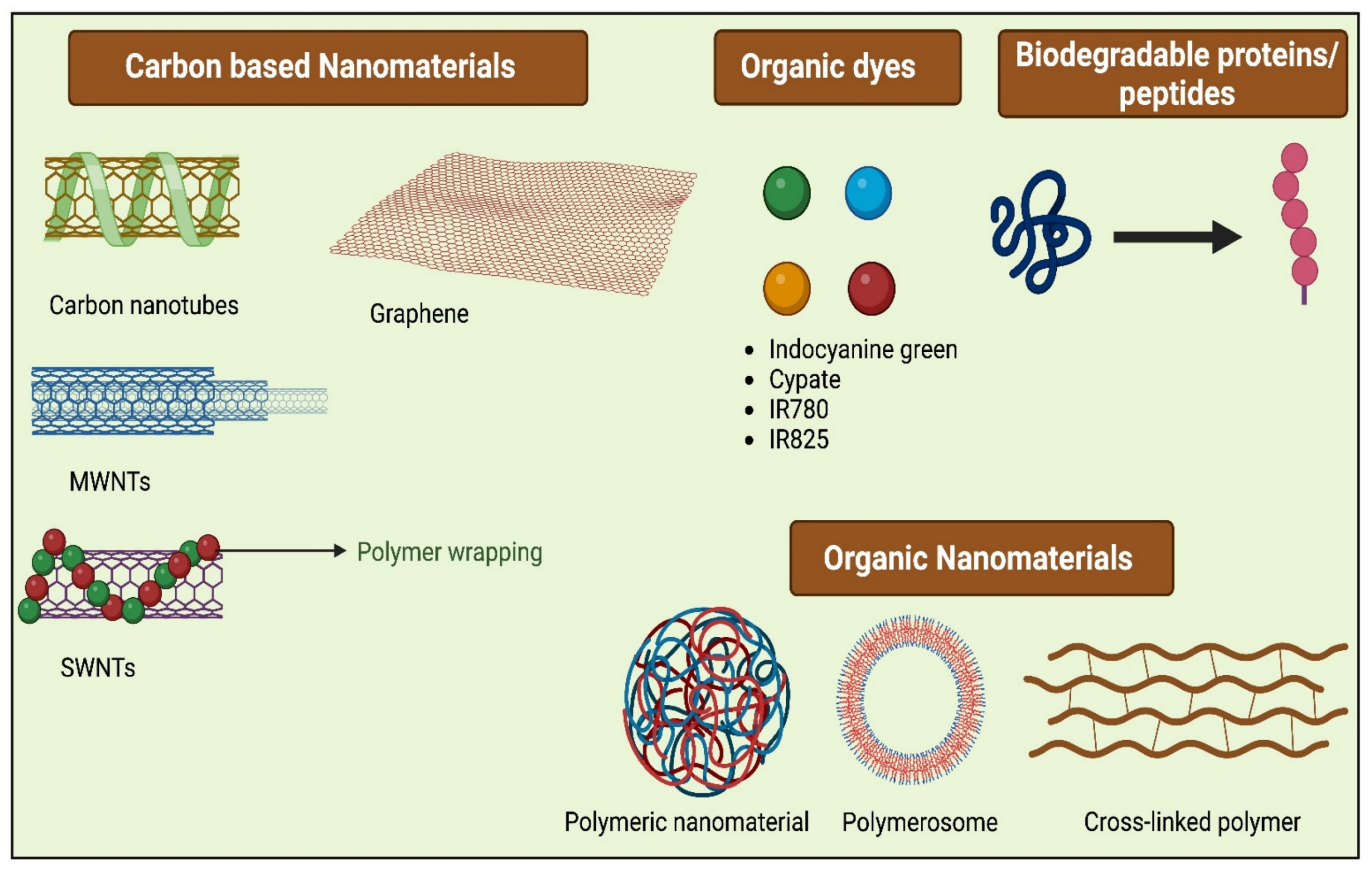
Different Bio-nanomaterials used for PTT.

The ideal PTAs possess characteristics like excellent photothermal conversion efficiency at a given wavelength and superior biocompatibility, which causes low toxicity to normal tissues, and they should possess excellent photostability. Typically PTAs are of different types (Figure 3), including organic nanomaterials (conductive polymers, NIR dyes), noble metal nanomaterials (Au nanorods, Au nanospheres, and others), metal compounds (iron compounds, copper, molybdenum, etc.), and carbon-based nanomaterials (carbon nanotube, graphene) 19. For the past few years, various inorganic PTAs have been synthesized and explored in *in vitro* and *in vivo* studies, which include metal-based nanocompunds such as Au, Ag, Pt, and transition metal-based sulfide, oxide, quantum dots, silica nanoparticles, as they exhibit surface plasmon resonance property leading to excellent NIR-light absorbance 20. Though these nanomaterials have excellent therapeutic efficacies in most cases, the complication lies in their non-biodegradability and cytotoxicity, which limits their application in clinical translation and therapeutics 21. Organic nanomaterials have better bio-compatibility than inorganic nanomaterials 22. To attain a greater photothermal effect with biocompatibility, biodegradability, low cytotoxicity, and early removal from the body, different bio-nanomaterials are synthesized, which are discussed in the following section also listed in Table 1.

### 3.1. Carbon-based Nanomaterials

#### 3.1.1. Carbon Nanotubes (CNTs)

These are cylindrical and composed of mono-layer sheets of carbon atoms, which are hybridized with sp^2^ molecules 23. CNTs have garnered significant attention in nanomaterial research due to their extraordinary electrical, mechanical, chemical, and thermal characteristics. These unique properties have propelled CNTs to the forefront of scientific investigation. CNTs are plasmonic nanoparticles with excellent thermal conductivity, due to which they penetrate the cells 42. CNT's thermal conductivity has several advantages, such as the produced heat being only restricted to the targeted part, preventing the off-target toxicity of tissues, and the temperature generated by heat being evenly distributed in the tumor cells. With the help of MRI temperature mapping, one can confirm the targeted tumor tissue volume 23.

Based on their arrangement of carbon sheets, two main types of nanotubes are present: single-walled and multi-walled.

##### 3.1.1.1 Single-Walled Carbon Nanotubes (SWNTs)

SWNTs are graphitic helical molecules that exhibit excellent physical and mechanical characteristics 24. Liu et al. found that SWNTs have good water solubility, low toxicity, and excellent biological stability 43. Liang et al. concluded that both *in vivo* and *in vitro* investigations demonstrated excellent stability and poor cytotoxicity of SWNTs. Adding polyethylene glycol (PEG) to SWNTs enhances the blood circulation time. Cy5.5, when added to SWNTs, exhibited Cy5.5-coupled SWNTs mediated PTT, resulting in systemic tumor ablation in mice due to the absorption at 808 nm. Cy5.5 enhanced the therapeutic index of PTT 44. Virani et al. introduced a novel therapy that treats superficial tumors and can prevent tumor recurrence. The treatment is predominantly a heat-based phenomenon which includes bladder tumor-specific SWCNTs that are inserted intravesically at a dose of 0.1mg SWCNTs/kg body weight by 30 secs treatment with a 360° NIR light for 24 hours, which affects the bound nanotubes. These nanotubes are directed to the tumor site via annexin V (AV) and phosphatidylserine 45. Lu et al. reported SWNTs having low toxicity, water solubility, bio-stability, and excellent photothermal conversion efficiency (PCE). Conjugating SWNTs with a dye further bound with antibodies was investigated for specific targeting of pancreatic tumors and to perform dyes imaging-guided cytotoxic PTT 46. Mei et al. designed selenium-based carbon nanotubes (Se@CNTs) with surface charge alteration and studied synergistic photo thermal-chemotherapy of triple-negative breast cancer (TNBC). When these internal Se@CNTs were irradiated, they led to the generation of reactive oxygen species (ROS), which induced the apoptosis of tumor cells. Also, their altered charge enhances the bio-responsive cellular uptake. This nanosystem ruled off the invasion and migration in other tissues of TNBC cells by managing the signal transduction pathways connected with it 23. Mckernan et al. combined PTT with targeted SWCNTs and immunostimulation with a checkpoint inhibitor to develop a novel treatment methodology for metastatic EMT6 breast cancer in syngeneic BALB/cJ mice. The newly developed formulation showed a remarkable synergy by enhancing the abscopal effect that relies on inhibiting cytotoxic T-lymphocyte-associated protein 4 (CTLA-4). As a result, the survival rate of mice significantly increased to 55%. Also, this treatment increased the CD4+ helper CD8+ cytotoxic T-cell count, which enhanced anti-tumoral effector cell activity to suppress tumor metastasis 47. Chen et al. altered SWNTs with an amylose derivative containing poly(L-lysine) dendrons (ADP@SWNT). This ADP@SWNT complex has pDNA transfection capacity and good water dispersion stability. ADP@SWNT/TNF blocks tumor growth and prevents metastasis *in vitro* and *in vivo*. NIR irradiation induces anti-tumor solid effects, suggesting it could be used in cancer therapy 24.

##### 3.1.1.2 Multi-Walled Carbon Nanotubes (MWNTs)

A few nanometers to a few micrometers in diameter, multi-walled carbon nanotubes (MWNTs) have emerged as prominent tumor drug delivery candidates, biological imaging, and photothermal tumor ablation. Furthermore, it is anticipated that MWNTs will absorb significantly more NIR radiation than other materials, such as SWNTs. After exposure to NIR, MWNTs may generate localized heat, leading to thermal annihilation of the tumor.

Zhang et al. used NIR irradiation as an external association with CREKA peptide as the targeting molecule, PEG as the shelter, MWNTs as the vector, and fibrin as the therapeutic agent, establishing a self-associated CMWNTs-PEG drug delivery system. This system had a promising tumor-targeting capacity with excellent PTT and clinical efficiency 25. Sobhani et al. first oxidized the CNTs (O-CNTs) and then wrapped the surface of CNTs with PEG to increase the permeability of MWNTs in water. This O-CNT-PEG showed lower cytotoxicity and reduced tumor size after PTT. Combining CNTs and Au-NPs showed plasmonic resonance with outstanding thermal conductivity and absorbance in the NIR region. In this case, the authors have fabricated MWCNTs with Au-NPs, which showed excellent efficiency in cancer treatment 42. Pathological examination of the liver, heart, kidney, and spleen showed no organ toxicity and good biocompatibility of these modified CNTs. Although the long-term safety issue of these molecules is still limited, these nano-agents have great potential in clinical and therapeutic applications 21.

#### 3.1.2 Carbon Dots

Carbon dots (CDs) have garnered significant research interest owing to their exceptional optical properties, biocompatibility, cost-effectiveness in production, and minimal environmental impact. These zero-dimensional nanocompounds exhibit desirable characteristics that make them highly promising for various applications. Their ability to turn light into heat has led to several new photothermal applications in recent years 48. They are known for their exceptional photostability, biocompatibility, thermostability, and photothermal conversion efficacy. CDs are an emerging class of photothermal agents. CDs could be used for imaging-guided PTT because they are fluorescent by nature.

Shinde et al. reported the development of soya lecithin-coated red fluorescent carbon dots (LRCDs) using leaves from *Clitoria ternatea* to develop hydrophilic LRCDs with improved bioavailability and theranostic properties. The resultant nanoparticle has a hydrodynamic diameter (HD d) of ~210 nm and can be used as a PTT agent in bioimaging and cancer therapy. PTT efficiency using 4T1 cells demonstrated a significant outcome, resulting in approximately 50% cell death following laser irradiation. This cell death was primarily attributed to the ablation caused by PTT and the cytotoxicity induced by reactive oxygen species (ROS). To check LRCDs' potential as a PTT agent in rupturing neo-blood vessels, CAM assays with chick embryos administered with LRCDs were reported 26.

#### 3.1.3. Graphene

Graphene-based nanomaterials have gained recent interest because of these compounds' distinctive structural, optical, thermal, electrical, and mechanical properties 49. Graphene is a 2D nanosystem of sp^2^-hybridized C-atoms organized in a hexagonal pattern 43. Graphene oxide (GO) and reduced graphene oxide (rGO) are used for PTT because of their characteristics, such as biocompatibility, simple formulating properties, modifiable surface structure, and high water solubility 23. Graphene is widely recognized as a highly effective nano-agent in PTT due to its exceptional optical absorption in the near-infrared (NIR) region 21.

##### 3.1.3.1 Graphene-Nanosheet

Graphene oxide (GO) nanosheet materials exhibit a broad absorption spectrum and excellent photothermal properties. Moreover, GO is a promising drug-loading platform due to its vast surface area. Liu et al. developed a novel formulation by using dopamine hydrochloride. A novel drug delivery system for cancer treatment was developed by synthesizing a composite material comprising polydopamine-cloaked mesoporous silica-coated reduced graphene oxide (rGO). Reduction and doping of GO with PDA showed twice the PCE of GO/MSN. This also exhibited the enhanced release of the drug in the tumor site 27. Yijuan Wang et al. performed a Monte Carlo-based docking study. Nanosheet is uniformly distributed on the surface of cancer cells. This caused a rapid temperature rise needed for tumor cell death 50. Lu et al. combined the rNGO sheets with MnO_2_ nanoparticles. Doxorubicin (DOX) and methylene blue (MB) are inserted into GO by a strong physical bond, which helps in enhancing the drug release under high temperatures 51.

##### 3.1.3.2 Graphene Oxide (GO)

Graphene oxide (GO) has several O_2_-based functional moieties. The functional groups in the molecules' basal plane are epoxy and hydroxyl groups, and the functional groups on the edge are carboxyl, carbonyl, hydroxyl, phenol, and lactone. These functional groups reduce Graphenes' hydrophobicity to a greater extent 43. Various types of GO nanoparticles used in PTT are listed in Table 2. GO allows the drug to bind through hydrophobic, H-bonding, π-π interactions, and electrostatic interactions due to its larger surface area 23. Liang et al. synthesized NCGO@DOX-FA NMs (Graphene oxide Nano complexes @ Doxorubicin- Folic acid) by loading nanocomposite (NGGO-FA) with DOX through π-π assembling and electrostatic interactions. It is effective for its large surface area, excellent drug loading capacity, prominent target specificity, good PCE, and photostability (irradiation at 808nm) 28. Chang et al. developed a flexible nano platform with BaHoF_5_ NPs adhered to GO. The formulated nanocomposite (GO/BaHoF_5_/PEG) is biocompatible and can penetrate lesions through the EPR effect. To perform PTT (irradiated with 808nm NIR laser) against cancer, NVP-AUY922 heat shock protein 90 (HSP 90) can be loaded into GO/BaHoF_5_/PEG 29. Qi et al. used aldehyde-modified PEG and carbonyl methyl chitosan (CMC) and formulated a Schiff base cross-linked hydrogel. This hydrogel can carry GO and needle-shaped nano-hydroxyapatite (HAP) inhibitors because of its self-healing and injectable capabilities. This treatment reduced the chemotherapeutic side effects and enhanced the fidelity of tumor diagnosis. It had a porous structure, better injectability, and self-healing characteristics 52. A controlled *in vivo* drug delivery system utilizing a nano hydrogel formulation comprising poly(N-isopropyl acrylamide) (PNIPAM) and graphene oxide (GO) was developed by Baipaywad et al. The introduction of PNIPAM/GO and PNIPAMAAM/GO into nano gel systems has significantly improved PTT effectiveness. By irradiating NIR light, DOX is released from the PNIPAM/GO-based Nano gel 53.

##### 3.1.3.3 Reduced Graphene Oxide (rGO)

rGO is a 2D carbon nanomaterial used as an efficient PTA in PTT due to its considerable PCE in the NIR region, more significant surface area, and functional groups for interaction with ligands and siRNA 54. rGO has two fold greater PCE than GO as a PTT agent 29. Table 3 details rGO types used in PTT. Zhang et al. identified a strategy for effortless loading of drugs into nano-sized nano-rGO for breast cancer PTT. Here, the drug used is Tamoxifen (TMX). The combinatorial TMX-nano-rGO composition exhibited considerable tumoricidal activity 30. Gai et al. identified that synthesized plant extract-based rGO has low cytotoxicity to cells. This rGO is used for PTT without further surface adjustments. When tested *in vitro* esophageal adenocarcinoma cell lines (OE-19), these resulted in the successful destruction of tumor cells 31. Liu et al. developed a nanocarrier (rGO@msilica) composite for synergistic chemo-PTT. It is a functional sandwich-like structure. The inner layer is made up of rGO, and the outer layer is made up of mesoporous silica. DOX that is loaded in this nano-composite is released smoothly into the acidic microenvironment. This nanocarrier works as a pH-induced drug nanocarrier and a NIR PTT agent 55. Lima-Sousa et al. used dopamine and rGO as reducing agents to produce PDOPA-rGO. The study found that PDOPA-rGO (polydopamine-reduced graphene oxide) exhibited strong absorption in the near-infrared (NIR) range and maintained its nano-sized dispersion within cells. To improve its colloidal stability, cytocompatibility, and physical and chemical properties, PDOPA-rGO was conjugated with thiol-terminated poly(2-ethyl-2-oxazoline) 42. This modified form of PDOPA-rGO showed promising characteristics and maintained its stability and compatibility within biological systems. Dash et al. conjugating IR780 photosensitizer to rGO, followed by the electrostatic interaction and coated with hyaluronic acid (HA). This can be taken by U87 glioblastoma cells. For multimodal cancer attack (CT/PTT/PDT), a pH-responsive nano system generated by the IR780-rGO/HA loaded with DOX was developed 56.

##### 3.1.3.4 Graphene Quantum Dots (GQDs)

GQDs are nano-sized structures with prominent edge effects and solid quantum characteristics that generate photoluminescence. GQDs are extensively applied in PTT using NIR-laser 49. Due to their high photothermal property and low toxicity, GQDs are a promising novel nanomaterial for PTT. Liu et al. reported GQDs having strong absorption at 1070 nm in the NIR-II region. The decomposition of H_2_O_2_ was tuned using a high magnetic field 32.

### 3.2. Small Molecular Organic Dyes

Molecular dyes exhibit excellent biocompatibility and superficial formulation characteristics. Molecular dyes can be reshaped smoothly according to our requirements to attain the desired product of absorption wavelength, specifically loading into tumor cells 58.

#### 3.2.1. Indocyanine Green (ICG)

The USFDA has approved ICG as a photosensitizer for clinical diagnosis. Under NIR laser irradiation, ICG can develop hyperthermia and generate ROS. However, several inherent limitations, like poor stability and rapid clearance rate, have controlled its widespread use as a PDT photosensitizer. Various nanocarriers, including polymeric NPs, calcium phosphate NPs, and mesoporous silica NPs, have been established to encapsulate ICG at a high loading rate 43.

Qing et al. developed pH-responsive hydrogels mPEG-luteolin-BTZ@ICG using bortezomib (BTZ) and indocyanine green (ICG) and tested on colorectal cancer by combining PTT/PDT with chemotherapeutic agents. Due to the combined effect of BTZ and ICG, tumor cells were effectively destroyed in both *in vitro* and *in vivo* studies. Combining chemo and laser treatment exhibited biological safety and paved a new path for treating advanced colon cancers 33. Xue et al. developed a multifunctional nano platform (FPPI NPs) by combining the ICG and magnetic prussian blue NPs with cationic polyethyleneimine. ICG particles are absorbed onto the surface of the prussian blue nano-shell, forming fine aggregates, which provide cellular ingestion of the drugs. The combination showed effects like tumor destruction by enhancing apoptosis, lowering toxicity, and improving histocompatibility and hemocompatibility 59. ICG acts as an effective photosensitizer by upscaling the ROS production. It also enhances the photothermal effect by peculiar absorption of near-infrared light. These ICG characteristics are coupled with tumor cell destruction 60. Wang et al. inserted ICG within SiO_2_-NH_2_ NPs, altering the NP's surface potential. The targeting capacity of SiO_2_-NH_2_@ICG NPs increased, making it stable enough to perform the photothermal conversion. After surface coating of ICG, these SiO_2_-NH_2_@ICG NPs absorption in NIR-spectra progresses widely. This study revealed that these NPs exhibit significant killing of tumor cells 34. Miranda et al. illustrated that the straightforward combination of indocyanine green (ICG) with liposomes containing DOTAP (1,2-dioleoyl-3-tri-methyl-ammonium-propane) leads to complete binding of ICG to the liposomes, thereby augmenting the optical properties of ICG. The amalgamation of ICG with liposomes not only amplifies optical properties but also demonstrates notable retention within 4T1 memory tumors upon intratumor injection. This retention is evaluated through fluorescence and photoacoustic imaging techniques, highlighting the effectiveness of the combined formulation 61.

#### 3.2.2. Cypate

Cypate is a bis-carboxyl-containing indocyanine green derivative that emits fluorescence and generates heat when exposed to NIR radiation. Yin et al. used the membranes of mesenchymal stem cells (MSCs) that are furnished with Fe and cypate for coating polymethacrylic acid (PMMA) nanoparticles. Cypate is an alternative to indocyanine green (ICG) to formulate Cyp-PMAA-Fe@MSCs. This is used for diagnosing lung cancer because of its high stability, high *in vitro*/*in vivo* PCE, and tumor accumulation. PTT-enhanced radiotherapy and MRI bimodal imaging/fluorescence are performed with Cyp-PMAA-Fe@MSCs 35.

#### 3.2.3. IR-780

The hydrophobic heptamethine IR-780 dye has a higher fluorescent intensity than the FDA-approved indocyanine green (ICG) dye. Still, it is less soluble in aqueous solution and more rapidly eliminated from the body. Encapsulating the IR-780 dye in amphiphilic micelle nanoparticles is anticipated to increase its aqueous solution stability 48.

The lipophilicity of biomolecular extracts limits the likelihood of increasing the therapeutic potential of several medicinal plants. Pemmaraju et al. conducted a study in which they employed Poly D, L lactic co-glycolic acid (PLGA) as a carrier to encapsulate and disperse a hydrophobic extract derived from Anthocephalus cadamba (CFAc). They also incorporated IR-780 dye into the formulation. This approach aimed to enhance cells' absorption and improve the CFAc extract's bioavailability to develop an effective cancer therapy. IR-780/CFAc (PCIR) NPs observed to HD d of 177.3 ± 85.5. When used at 20 µg/ml concentrations, PCIR NPs showed increased cytotoxicity and selectivity towards malignant cells, killing 51.2% of B16 cell lines 36. Tutty et al. used IR-780 dye-loaded liposome (Liplmage ^TM^815) for deep tissue imaging and found no DNA damage or cytotoxicity 2.

Other than IR-780, many other dyes also generate photothermal effects. Pebam et al. prepared a polymeric nanosystem using a combination of IR775 (a hydrophobic NIR dye), and ethyl acetate extract of *Terminalia chebula* (EAT) encapsulated in polylactic acid nanoparticles (PpIR NPs). Cellular infiltration was found to be increased in a lung cancer cell line (A549). Cytotoxicity studies indicated PpIR NPs inflicting cell death of ~82.46 ± 3% upon NIR irradiation via ROS generation and resultant hyperthermia. Further qualitative and quantitative analyses revealed downregulated PARP and HSP70 and upregulated γ-H2AX, mutually enhancing cancer cell death upon PpIR NPs treatment with NIR light irradiation 62. To enhance the mucoadherence potential by inhibiting/lowering the mucin expression in ECM of breast cancer, Jogdand et al. reported a chitosan-coated non-ionic surfactant-based bilayer vesicle (Niosome) as a carrier for IR 806 dye (NioIR-C NPs). The nanosystem has an HD d of 99.5 ± 42 nm and is shown to cause extended hyperthermia-induced ablation up to 93% against MCF-7 cells upon NIR exposure 63.

### 3.3. Proteins/Peptides-based Nanomaterials

Proteins and peptides have become superior delivery agents for different diagnostic or therapeutic components due to their biodegradability, biocompatibility, and good water solubility. Nanomaterials can be directly synthesized by using them as bio templates. On the surface of proteins/peptides, many carboxylic and amino groups are present, which interact with other functionally active substances like NIR-dyes, targeting ligands, photosensitizers, and various imaging agents to attain multifunctional diagnostic and theranostic outcomes 7. In another study, bimolecular hydrogels are developed to self-assemble bio-molecules (proteins, peptides, polysaccharides). These are used in PTT, PDT, and other treatments. These hydrogels can target drug delivery, enhance drug release, increase efficiency, and reduce cytotoxicity. However, these hydrogels have some limitations, like deep penetration of pH and enzyme-degraded hydrogels required and a better understanding of tumor immunity 64. Meng et al. combined HAS (human serum albumin) with PTX and ICG and developed stable NPs in an aqueous medium. PTX-ICG-HAS NPs concentrated in the tumor site by EPR effect, and exhibited removal of 4T1 subcutaneous tumor without any toxicity or regrowth of tumors 37.

### 3.4. Polymer-based Nanomaterials

To target early-stage retinoblastoma (Rb) tumors since they are less lethal and do not obstruct large portions of the visual field, Mudigunda et al. reported a multifaceted polymeric nanoparticle (PNPs) with two hydrophobic components: Palbociclib (PCB) and IR820 (IR). The PCB/IR PNPs with an HD d of 176 ± 43.8 nm had significant cytotoxicity (86.5 ± 2.3%) in Y79 cell lines. The PCB/IR PNPs exhibited a steady rise in temperature from 28.3 ± 0.5 to 49.9 ± 0.8 °C under NIR light, accounting for the controlled release of PCB, and had a long-lasting impact on tumor cells 65. Appidi et al. reported PDPC NPs, a lipo-polymeric hybrid nanoparticles surface coated with Au (PDPC-co-loaded with IR780 (PDPC-IR-Au NPs) with HD d of 94.2 ± 14.9 nm. The nanosystem exhibited plasmon-enhanced fluorescence, demonstrating a heightened intracellular ROS, inducing DNA damage and apoptosis when tested against MCF-7 breast cancer cells 66.

#### 3.4.1. Conducting Polymers

Conducting polymers (PANI and PPy) are used widely for biomedical and bioelectronic applications. The optical absorption quality of conducting polymers made them accessible in PTT. Due to their low cost and superior biocompatibility to inorganic nanomaterials, they are widely used as ablation agents for PTT. Upon laser irradiation, a bipolar product eventually degrades into a photon band and generates heat 67. Liu et al. harmonized an exceptional polymeric PTA that is obtained from poly-(3,4-ethylene dioxythiophene):poly-(4-styrene sulfonate) (PEDOT:PSS) for cancer PTT. PEDOT:PSS is a conducting polymer derived from polythiophene with specific absorbance in the NIR range. To improve PEDOT: PSS biocompatibility, it is linked with PEG. The administration of PEDOT:PSS-PEG (200µl, 1mgmL^-1^) tailed by NIR-laser (808nm, 0.5 W. cm^2^, 5 min) irradiation caused a temperature rise from 31-51^0^C in target tissue sites resulting in tumor destruction. This nanoformulation also showed tumoricidal activity against 4T1 tumor cells in mice 68. Chen et al. proposed and formulated double-acceptor conjugated polymers (TTQ and DPP), which showed NIR absorption at 1064nm and fluorescence emission around 1200-1400nm by managing the molar ratio of two acceptors. Nanoprecipitation of these conducting polymers resulted in the production of different nanoparticle combinations (P1 NPs, P2 NPs, P3 NPs), of which P1 NPs were tested in 4T1 tumor-carrying mice because of their significant NIR-II F1 signals, higher biocompatibility, more excellent photostability, and better photothermal capacity. Hence, it has an immense application in eradicating tumors 69.

##### 3.4.1.1. Polyaniline (PANI)

Polyaniline (PANI) is the first polymer-based photothermal anticancer agent to be reported. Huang et al. constructed a virtuous hydrogel (GG@PANI(Fe)-borax) by doping iron with polyaniline [PANI(Fe)] that is attached with guar gum (GG) moieties by borate/didiol bonds. This study first applied Guar gum chains as a primary construction unit. GG@PANI(Fe)-borax exhibited rapid self-healing properties, good injectability, and amendable sol-gel transformation under pH or thermal stimulation. Iron-doped PANI generated free ^º^OH radical in the H_2_O_2_-enriched tumor microenvironment. Hydrogels show excellent photothermal properties and controlled release of drugs in the NIR-triggered range, aiding in the development of synergistic PTT, chemo-dynamic therapy, or chemotherapy used in *in vitro* and *in vivo* melanoma conditions 38. Wang et al. developed an effective photothermal theranostic agent of Au nanostar@polyaniline core-shell nanomaterial, which possesses a tremendous PCE on low-dose administration for PAI (photoacoustic image)-guided PTT. This PTA is modified by hyaluronic acid (HA) without affecting its photothermal efficiency. AuNSPHs show outstanding biocompatibility in PAI-guided PTT for the ablation of tumors 39.

##### 3.4.1.2. Polypyrrole (PPy)

Organic polypyrrole (PPy) has high PCE and enhanced biocompatibility characteristics. Cheng et al. developed PPy(polypyrrole)-CTD(cantharidine)@MIL-100@MPCM(coated macrophage cell membranes) NPs (PCMM NPs) and succeeded in improving the rate of Fenton reaction, which is based on PTT. This causes the release of PPy cantharidine and iron ions in the PCMM NPs 70. PPy is photosensitive in the near-infrared region. Xu et al. developed polypyrrole (PPy)-poly (ethylene imine)-silk nanoparticle [PPR_ILK_]. This formulation was tested in combined photothermal and gene therapy. It caused lymphatic metastasis inhibition and lowered the invasive ablation of the tumors 40.

#### 3.4.2. Melanin-like Polymers

It is a polymer found in various plants, animals, and human beings with antioxidant, radio-resistant, and anti-neoplastic properties. Melanin also possesses intense radiation in the NIR range, which benefits PTT 68. Zhang et al. reported a bio-acceptable strategy in which melanin PEGylated nanoliposomes showed effective clinical translation and theranostics with good biocompatibility 41. Du et al. reported Melanin-like PDA compounds and polyphenol-based PTAs, which are studied for cancer-PTT and combination therapies due to their biodegradability, biocompatibility, and ability to be synthesized sustainably and scalable 9.

### 3.5. Microspheres of MXene for PTT

In 2011, Naguib et al. discovered MXene 1, a novel 2D transition metal. MXenes are produced by printing the parent MAX phase to exclude the A-group element (Aluminium). The large surface area, ease of functionalization, and excellent photothermal conversion efficiency (PCE) of 2D MXenes make them highly promising compounds for clinical applications in biomedical research. These unique properties of MXenes enable their potential use in tumor cell PTT 3.

Perini et al. examined the effect of Ti3C2Tx MXenes mediated-PTT on two distinct 3D breast cancer models. In both models, an IC50 concentration of 50 g/mL was used for the PTT. There was a significant decrease in cell sustainability and an increase in ROS after PTT. In both 3D models, they also examined the effect of PTT on macrophage and endothelial cell migration towards cancerous regions. They found that PTT-mediated MXenes downregulated tumor progression by raising the temperature in the bio-printed model 4.

### 3.6. Naphthalocyanines (Nanonaps) and Phathalocyanine

Zhang et al. strategically engineered surfactant-stripped nanoformulated naphthalocyanines (nanonaps) to possess robust absorption in the near-infrared (NIR) range, capitalizing on its ability to minimize light scattering and absorption by native biological tissues. Through the incorporation of Pluronic F127 and employing low-temperature membrane processing, they achieved the formation of dispersed frozen micelles characterized by exceptional contrast in the NIR region. In our study, we present the versatility of nanonaps for multifunctional cancer theranostics, encompassing applications such as lymphatic mapping and comprehensive photoacoustic imaging of entire tumors post intradermal or intravenous administration in rodents.

Notably, pre-formed nanonaps exhibited intrinsic properties suitable for positron emission tomography, passively accumulating in subcutaneous murine tumors without requiring further modification. Leveraging the unique light absorption capabilities of nanonaps beyond the visible spectrum, we devised a topical upconversion skin cream for anti-tumor photothermal therapy. This innovative approach enables laser placement guided by the naked eye, showcasing the potential for targeted and effective cancer treatment. Porphyrin and phthalocyanine molecules exhibit promising potential for utilization in both multimodal imaging and therapeutic applications^71^.

Phthalocyanine stands out as a prominent organic photothermal agent, boasting a well-defined chemical structure, reproducible synthesis, and robust absorption in the near-infrared region. Its photophysical and photochemical properties are easily tunable, adding to its versatility. Furthermore, the compound exhibits inherent biodegradability, contributing to its environmental friendliness^72^.

Pan et al., A novel cruciform phthalocyanine pentad has been innovatively designed, synthesized, and introduced for the first time. The resulting water-soluble nanoparticles (Zn 4-H2Pc/DP NPs) are formed through the assembly of this unique molecular material, aided by DSPE-PEG 2000-OCH3. These nanoparticles showcase distinctive absorption properties in the NIR-II region, specifically at 1064 nm, featuring a substantial extinction coefficient of 52 L g-1 cm-1. Moreover, they demonstrate a remarkable photothermal conversion efficiency of 58.3% and generate an intense photoacoustic signal^73^.

## 4. Combinatorial Effect of PTT with Cancer Therapies

**Figure 4:**
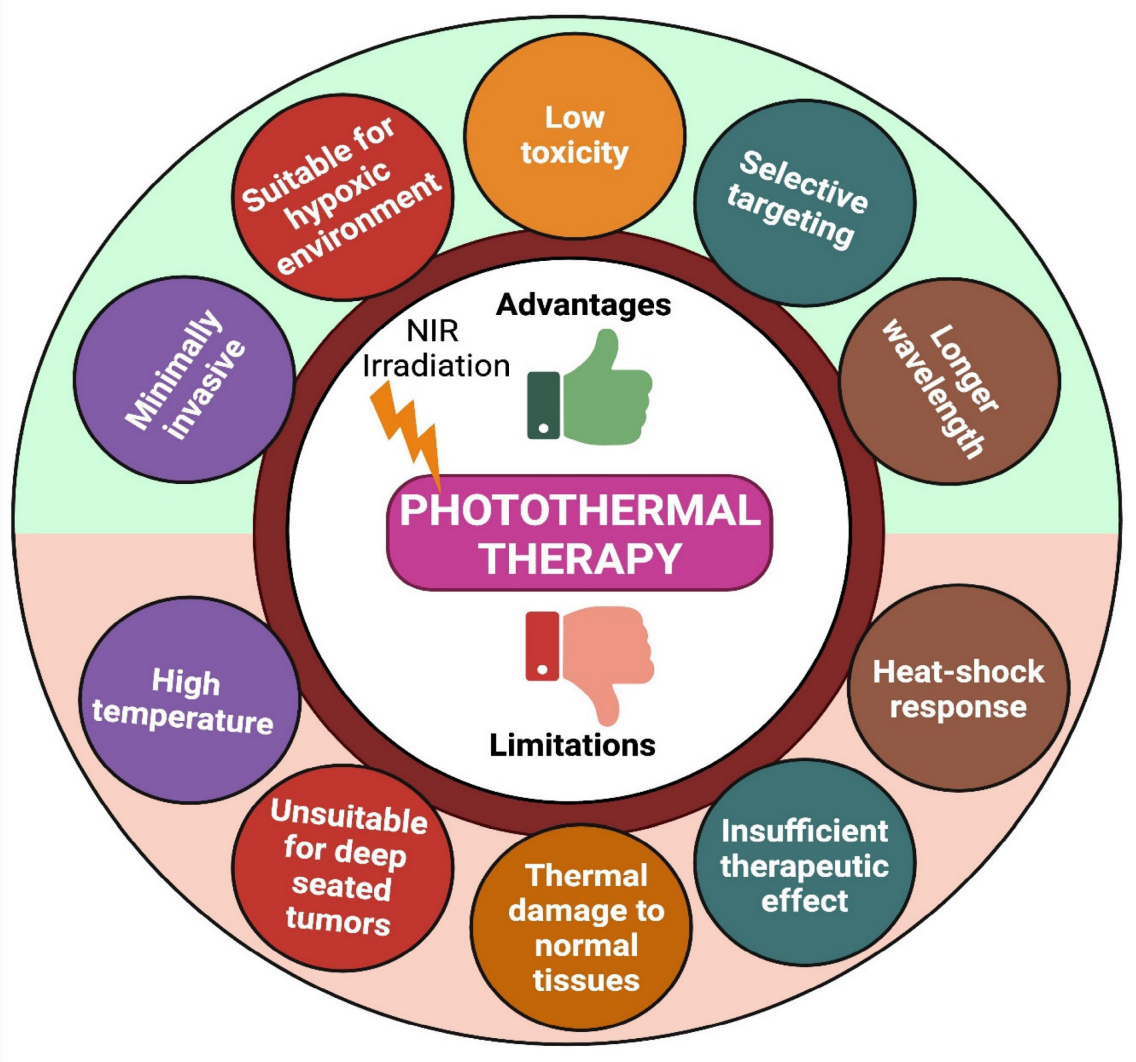
Advantages and limitations of PTT.

Due to higher therapeutic efficacy, PTT has emerged quickly over the last few decades. However, photothermal treatment still has many limitations (like high temperature, limited penetration depth of light, thermal damage to normal tissues, and heat-shock response) that block its usage in many clinical conditions (Figure 4) 74. To overcome these limitations, we need combination therapy- which means concurrently co-targeting drugs or synergizing therapeutic approaches in different forms for efficient cancer therapy. Indeed, combination therapy has a significant advantage in enhancing the prognosis of many diseases 33.

### 4.1. PTT Combined with Chemotherapy (CHT)

Chemotherapy (CHT) is broadly considered a thriving cancer treatment in clinical translation for metastatic and advanced-stage tumors. CHT has also received outstanding achievements in clinical translation for increasing the life expectancy rate of many cancer patients because of its extreme reliability, efficiency, and convenience of use. Apoptosis is one of the mechanisms by which CHT kills the tumor cells. However, some limitations, like the limited amount of dosage, non-specific distribution, high level of cytotoxicity, and low drug penetration in the tumor regions, are the primary reasons for the poor prognosis of this therapy in cancer patients. To lower these side effects, a combination of CHT with PTT has been adapted for better results since PTT possesses efficient intracellular drug delivery, sustained release of the drug, enhanced drug retention in the target site, and block drug resistance properties 16.

Liu et al. developed In aqueous solution, maleimide-based enediyne, and PS IR820 co-assembled to form nanoparticles [EICN (carrier-free DDS (drug delivery systems)] by combining maleimide-based enediyne and IR780which leads to a synergistic PTT/CHT activity. EICN is enriched with the acid/NIR, which causes free radical generation, drug release, DNA-breaking capacity, and dual-responsive degradation due to the co-binding of enediyne and IR780. The diameter of EICN was 90nm, and it is highly stable. Hence, this carrier-free DDS with dual acid/NIR-responsive EICN is an excellent therapeutic agent for CHT/PTT 75. Li et al. generated a new type of nano agent when the NIR laser was irradiated on a polymeric nano micelle (PT@MFH- polymeric nano micelle-based nano agent). This nano agent delivers the chemotherapeutic drug Paclitaxel (PTX). Site-specific hyperthermia was rapidly achieved via 808 nm laser irradiation. Since the drug is delivered between the "silent state” (before photo triggering) and the “activated state” (after photo triggering), it provides a great contrast in local temperature. This rise in temperature and site-specific drug delivery set a great foundation for synergistic CHT and PTT in tumor treatment and excellent therapeutic efficiency 76.

The stimulus-responsive drug delivery and controlled release applications are very interested in the reduced graphene oxide (rGO) nanosheet modified with proteins. Ah Cheon et al. developed a novel approach to fabricate functionalized reduced graphene oxide (rGO) nanosheets by incorporating bovine serum albumin (BSA) and loading them with doxorubicin (DOX), resulting in DOX-BSA-rGO nanosheets. They used UV-visible spectrophotometer and X-ray photoelectron spectroscopy (XPS) to analyze how BSA-functionalized GO nanosheets broke down and how well they loaded drugs. DOX-BSA-rGO nanosheets were taken up by cells in a dose-dependent way, but they did not kill the cells. Recent studies have revealed a promising treatment approach for brain tumor cells utilizing DOX-BSA-rGO nanosheets, which employ chemo-PTT activated by near-infrared (NIR) light. 77.

### 4.2. PTT Combined with Immunotherapy

**Figure 5:**
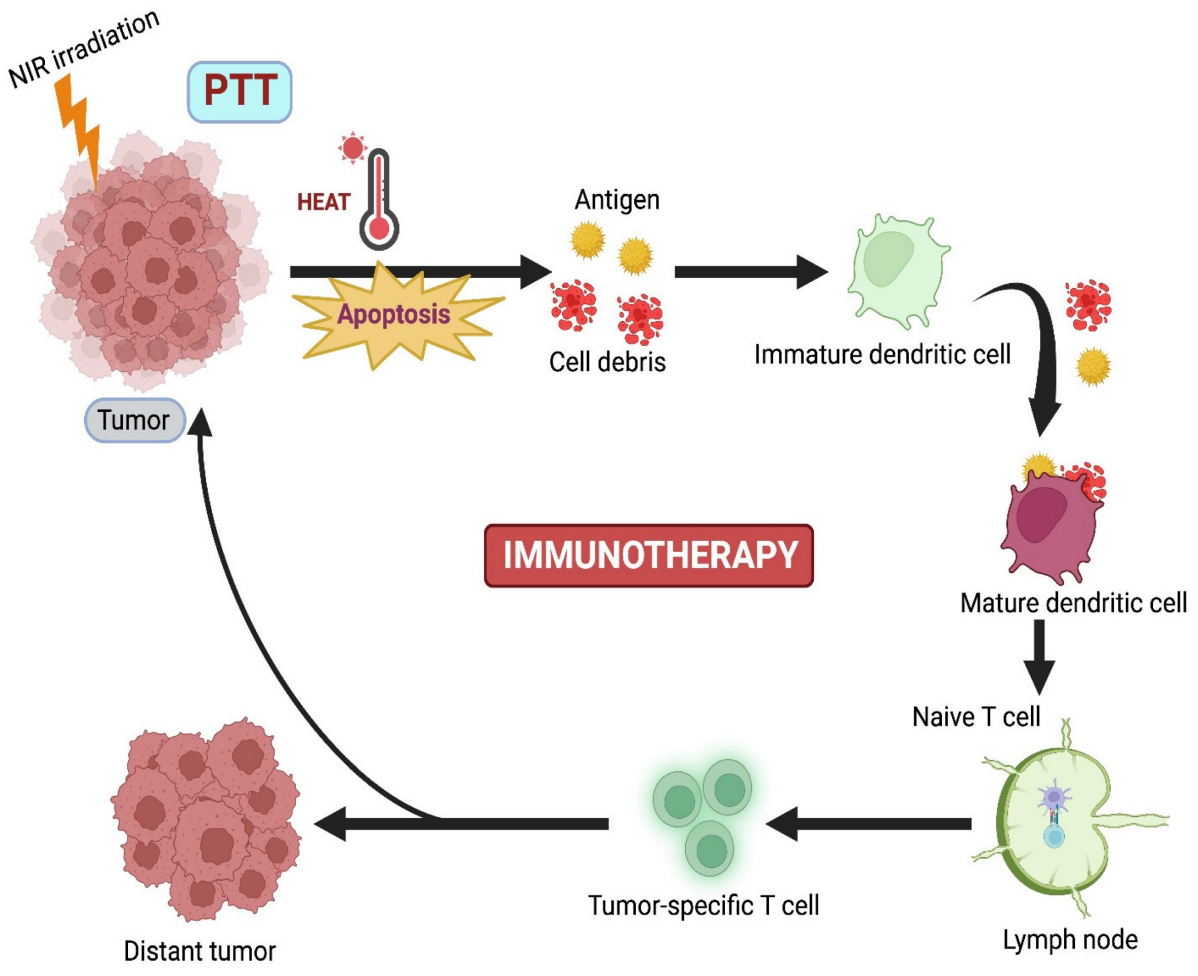
PTT combined with Immunotherapy.

Although PTT has lower tissue invasiveness, it is limited by the absence of prolonged efficiency against the regrowth of tumors, causing cytotoxicity due to the slow biodegradation of PTT agents 78. Immunotherapy (IMT) is currently emerging as a promising curative approach that activates the host's immune response against the tumor. Nevertheless, some disadvantages are also present in IMT, such as poor immunogenicity, mutation rate, inappropriate host immune response, and low penetration of immune cells. Based on these drawbacks, combination therapy, where PTT-induced IMT, develops innate and adaptive immune responses against cancer (Figure 5) 16.

Balakrishnan et al. proposed that immune checkpoint blockade with monoclonal antibodies like CTLA-4 and PD1/PDL 1 has upgraded clinical outcomes for cancer patients. Here, a combination of PTT with ICB caused both immunogenicity and tumor-specific cytotoxicity. These two therapies complemented each other and potentially enhanced antitumor effects 79. Liu et al. combined PTT with a novel localized ablative immunotherapy (LAIT) for intra-tumor targeting of immunostimulant N-dihydrogalactochitosan (GC) for the treatment of breast tumor-Ag 9MMTV-PyMT. They used single-cell RNA to compare transcriptional changes induced by PTT, GC, or PTT+GC in B-cell tumor microenvironment (TME). They concluded that PTT, GC, or PTT+GC enhanced the expression of genes required for B-cell activation 80. Liu et al. combined gellan gum and Dawson-type (P_2_Mo_18_) polyoxometalate (POM) and developed an implantable hydrogel system (R848/POM@GG) against resiquimod (R848) for synergistic PTT-IMT of cancer. Gellan gum hydrogel prepared from POM(POM@GG) showed excellent photostability and good PCE (63.1%). R848/POM@GG had an excellent tumor ablation rate of 99.3% and minor metastasis in mice breast tumors. R848 enhanced the generation of TNF-α, IL-2, and IL-6, developing a solid antitumor immune system against cancer 78.

### 4.3. PTT Combined with Radiotherapy

**Figure 6:**
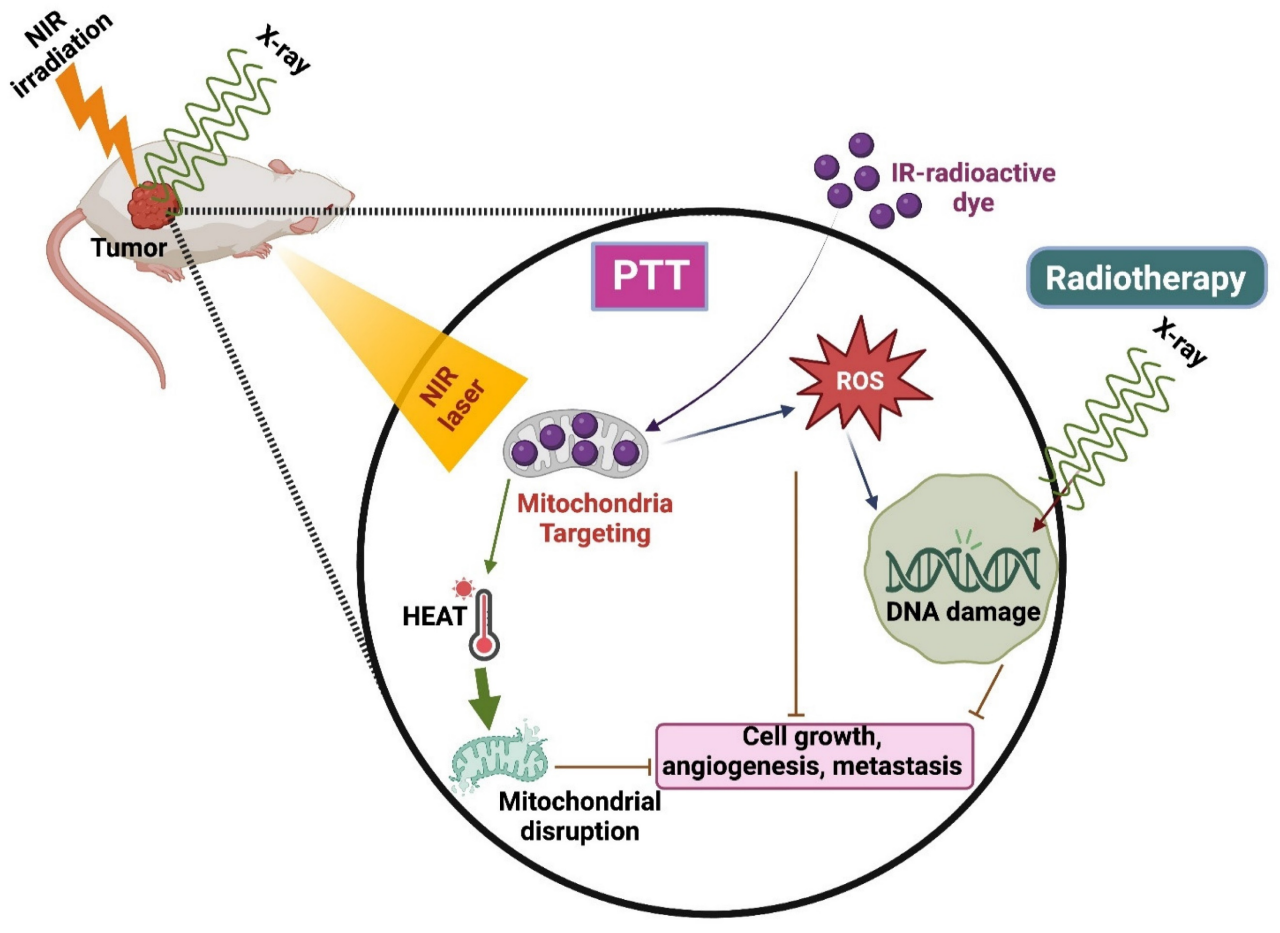
PTT combined with Radiotherapy.

Radiotherapy is the commonly used technique integrated with radio sensitizers for better results in clinical translation 81. It has a few limitations, such as causing damage to healthy surrounding tissues and the inability to kill the cells that are not visible in the imaging scan zone. Henceforth, combining RT with other therapies, especially PTT, is highly needed to increase RT's efficiency (Figure 6) 82.

A nano-phototherapeutic system was developed by Liu et al., where they combined poly (thiodiethylene malonate) (SPSDEM) and PEG-PSDEM-PEG, both loaded with suberoylanilide hydroxamic acid (SAHA), to enhance radiosensitivity. It presented the ability to perform morphological changes and induce therapeutic effects against X-ray radiotherapy and NIR irradiation by combining ROS-induced sensitization of RT and photo-mediated reactions. The unloading of SAHA triggered by ROS and the increase in vascular permeability induced by hyperthermia sensitize the target tissue in radiation therapy. This enhanced sensitivity results in improved therapeutic outcomes when combined with photothermal therapies. This combinatorial ROS-induced sensitized RT causes the breakage of double-strand DNA and blocks cell proliferation in 4T1 breast cancer cells. Due to this combination therapy, a reduction of cell viability was observed when compared to RT alone 81. IR-83, a multifunctional bioactive small molecule, exhibits preferential tumor accumulation, near-infrared imaging, and RT/PDT/PTT effects. It was constructed by 2-nitroimidazole (radio sensitizer) in the core of heptamethine cyanine dyes, having intrinsic tumor penetration and phototherapeutic efficiency. IR-83 was aggregated in the tumor site, preventing tumor growth and metastasis by integrating RT/PDT/PTT. Studies revealed that laser irradiation induced ROS production and heat generation after the accumulation of IR-83 in tumor cells 82.

### 4.4. PTT Combined with Gene Therapy (GT)

In GT, gene vectors enhance the clinical usage of nucleic acids (DNA, siRNA, mRNA, miRNA) 83. However, GT also has limitations like safety concerns of viral vectors, failed clinical trials, and unwanted immune system reactions. To overcome these problems, a combinatorial approach involving PTT with GT has been implied to attain positive results.

Odda et al. constructed a unique photothermal nanocarrier that has been synthesized for combining GT and PTT of cancer cells. In this, they combined surface-modified iron oxide (α-Fe_2_O_3_) NPs with poly (3,4- ethylene dioxythiophene) (PEDOT) polymer, which is coated onto the surface by a one-pot in-situ oxidative polymerization method. Core-shell α-Fe_2_O_3_@PEDOT NPs have a uniform particle size and show the surface character of positive charge, permitting it to uptake by siRNA Bcl-2(B-cell lymphoma-2) for loading into breast cancer cells. These core-shell α-Fe_2_O_3_@PEDOT NPs have exhibited excellent biocompatibility and water dispersibility and have powerful NIR irradiation compared to α-Fe_2_O_3_ NPs. They showed better PCE (η=54.3%) and good photostability in the region. Results revealed that GT/PTT combined therapy demonstrated excellent tumor cell apoptosis when compared to alone GT or PTT 84. Liu et al. synthesized hyaluronic acid-coupled Au nanorods by ring-opening polyglycidyl methacrylate with ethylene diamine for photoacoustic image-guided combinatorial PTT-GT. This is expected to enhance the circulation time of nanoformulation in the body. Au nanorods improve the composites through their PAI function or photothermal effect. These composites exhibited biocompatibility, stability, effective targeting of tumor tissues, and a more extended retention period in the body 85.

### 4.5. PTT Combined with PDT

**Figure 7:**
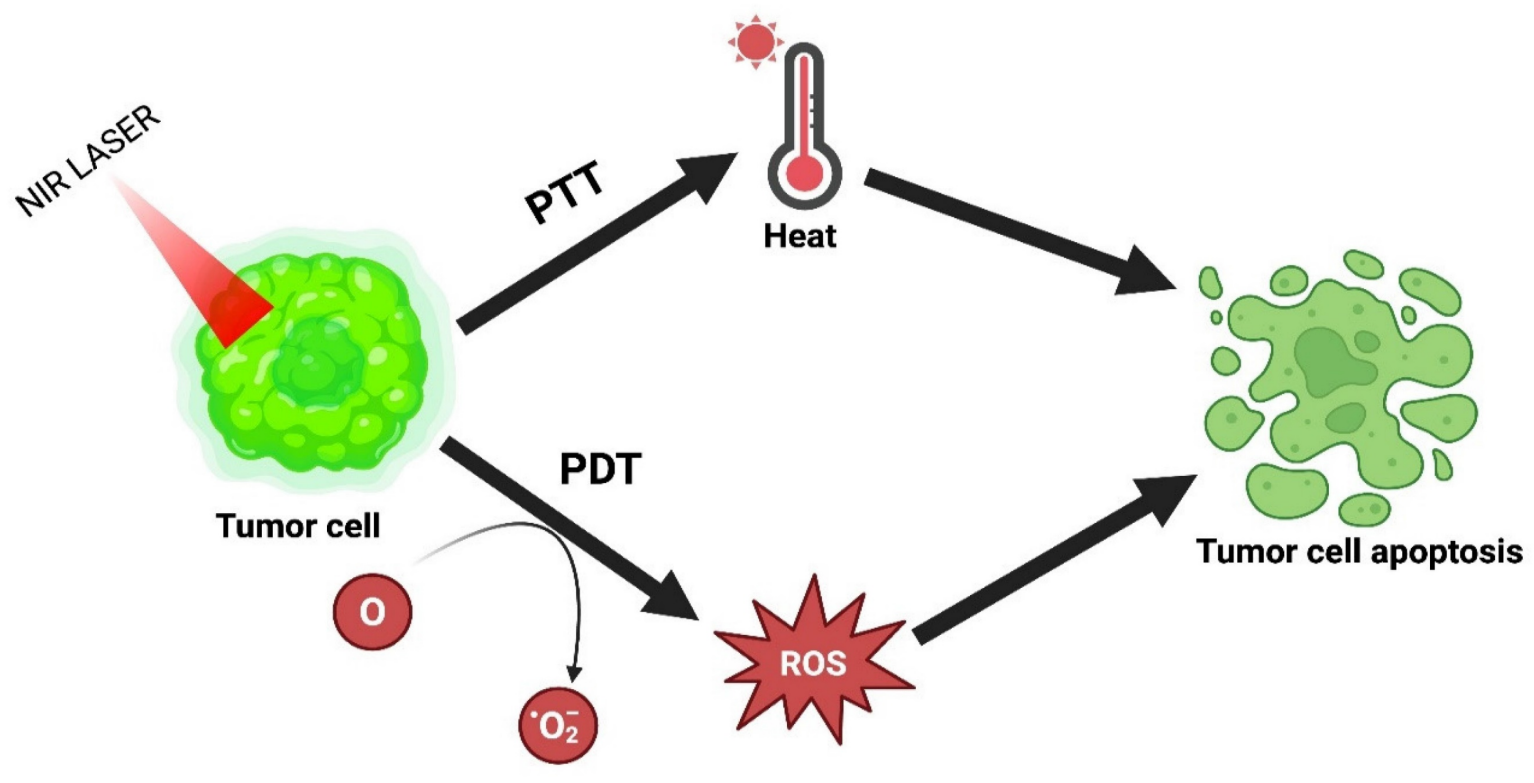
PTT combined with PDT.

PDT has emerged over the last few years as an alluring alternative treatment. Under laser irradiation, it generates ROS by using photosensitizers to influence the killing of cancer cells. This therapy has several advantages: high selectivity, lower invasiveness, excellent curative properties, and fewer side effects. However, its use in clinical translation is restricted because of tumor hypoxia or lower tissue penetration. The combinatorial effect of PTT with PDT resolves this problem and achieves the desired outcome 16. The combinatorial mechanism of PTT and PDT is explained in Figure 7.

Rajalakshmi et al. proposed multifunctional chlorophyll-rich fluorosomes produced from Spinacia oleracea co-assembled on a polydopamine core (SPoD NPs) to contribute to improved photothermal transmission. SPoD NPs presented a blend of PDT/PTT that checked both normoxic and hypoxic cancer cell growth. SPoD NPs were 176 ± 10 nm with enhanced physiological stability and tumor regression by synergistic PTT and PDT. To efficiently target the tumor and to overcome metastasis, resistance, and angiogenesis, the MCF-7 cell spheroids were treated with SPoD NPs, markedly showing profound damage to the center of the spheroids 86. Alvi et al. reported an alternative therapeutic approach with liposomal Au nanoparticle encapsulating curcumin (Au Lipos Cur NPs) to address the recurrence of acne subjected to antibiotic resistance. 100-120 nm in size using dual light-mediated therapy. These nanoparticles exploit the localized follicular delivery by exerting a positive zeta by iontophoresis, causing thermal ablation of sebaceous glands and inhibition of bacterial growth via hyperthermia and ROS generation when irradiated with NIR (808 nm) and blue light (dual light) 87. Li et al. successfully constructed ICG@MoS_2_ NPs. This formulation attains the combinatorial effect of PTT-PDT and successfully inhibits p-glycoprotein (P-gp) protein. P-gp is a membrane protein encoded by the MDR1 gene at the ATP-dependent efflux pump in various cancer cells. This pump can induce multidrug resistance (MDR) by eliminating the antitumor drugs from the cell, thus causing the inhibition of P-gp, thereby increasing the PDT effect 88. In their study, Guo et al. formulated a new approach by synthesizing a hybrid material using a combination of folic acid and chlorin e6 functionalized graphene oxide (GO). This formulation was designed to enable a synergistic approach using PTT and photodynamic therapy (PDT). Interestingly, the composition exhibited a remarkable ability to penetrate macrophages effectively. The combinatorial effect of PTT-PDT was observed to have greater killing efficiency towards cancer cells 89. By facile two-step method, Liu et al. synthesized ICG-encapsulated hyaluronic acid surface coated with polydopamine nanoparticles (IPPH). These synthesized NPs exhibit potent PCE and production of single oxygen radicles. Studies showed that IPPH can inhibit tumor growth by the combinatorial effect of PTT-PDT. IPPH NPs exhibited excellent potential to use a new photothermal-photodynamic molecule for cancer treatment 90.

## 5. Effect of LED in PTT

Recent research has shifted its focus towards LED (Light Emitting Diode) light sources for PTT due to their affordability and reduced side effects. LED-based PTT offers a more accessible option for research laboratories and clinical facilities 91. In the past, the treatment of tumors primarily relied on the use of tightly focused, synchronized, and highly uniform beams of light with the potential for high power densities. However, recently, there has been a shift towards utilizing non-coherent light sources like light-emitting diodes (LEDs) and broad-spectrum lamps. LEDs offer several advantages, including the absence of laser safety concerns, convenience for home-based treatments, the capability to illuminate a larger tissue area simultaneously, the potential for wearable devices, and significantly lower costs per milliwatt light output 92.

Unlike the focused and concentrated laser beams in laser-based devices, LED light sources emit diffuse, broadly dispersed light that lacks concentrated hotspots. This characteristic mitigates potential hazards associated with laser light, such as eye injuries and tissue damage due to heat. The LED-based PTT offers an added advantage in delivering a greater overall light output than laser devices. LED lights' convenience and safety make them highly appealing for everyday use in residential settings 93. Utilizing an LED light source within the NIR spectrum may offer a more convenient and less hazardous PTT method than lasers. Operating lasers is a challenging and costly endeavor, and they are intentionally engineered without the capabilities of LED lights. In contrast, the latest LED light sources excel in providing higher intensity and longer-duration NIR light while mitigating the risks typically associated with lasers. Arrays of compact LED light sources can be engineered to illuminate a broader treatment area than lasers, enhancing their potential for practical clinical application 94.

## 6. Effect of Mild Hypothermia in PTT

Unlike the conventional laser treatment, due to poor reach to in-depth tissue and lower photo thermal conversion efficiency demands a long-term and higher intensity laser irradiation in order to realise the efficient tumour killing sustaining some non-specific injury to bystander cells (vasculature, cytoskeleton) and host anti-tumour immunity. Thus, harnessing mild temperature (42-45 °C) PTT and PDT therapeutic modalities either alone or in combinations via modulation of HSPs expression, ROS generation, overseeing autophagy and organelle specific tumour targeting have attracted certain attention in sensitizing cells against mild temperature rise, ultimately curbing cancer cell proliferation 95-97. Huang et al. loaded IR820 and an anti-PD-L1 antibody into a thermosensitive lipid gel depot that can undergo a gel-to-sol phase transition to remodel immunologically cold tumors into hot tumors. Using mild temperature (35-45 °C) tuned NIR-irradiation, upsurge in the recruitment of tumor-infiltrating lymphocytes, upregulation of PD-L1 on tumor cells, and upscaled T-cell activity against tumors were observed later supported by *in vivo* studies on mouse model, thus maximizing synergistic approach 89.

## 7. Conclusion and Future Perspective

Different types of selective bio-nanomaterials have been developed for PTT. Although the mentioned bio-nanomaterials have excellent selectivity, biocompatibility, biodegradability, and adequate biosafety, their long-term toxicity and side effects are under consideration. The present NMs used in PTT have low biodegradability and long-term cell toxicity. Hence, we need to synthesize nano-sized or biodegradable-NMs to improve metabolism, rapid degradation, and enhance elimination from the body to minimize the toxicity effects.

These NMs exhibit therapeutic properties by converting the photon energy into thermal energy. We can optimize the PTT ability of bio-NMs by modifying its external features such as laser irradiation on the tumor site, laser-light management (time of irradiation and power density), the retention time of the PTT agents in the body, as well as morphological features like size, shape and surface properties of nanomaterials. Furthermore, research is required to select an ideal agent for PTT and other synergistic therapies. So, bio-nanomaterials have a significant advantage over other nanomaterials because of their biocompatibility and biodegradation properties. In short, using appropriate PTT agents with biosafety, biodegradability, optical characteristics, and tumor-specific targeting in combination with PTT and other therapies such as PDT, GT, RT, CHT, and IMT leads to better results for cancer treatment.

PTT boasts numerous advantages, including precise control over both time and space, cost-effectiveness, and minimal invasiveness. Furthermore, PTT stands out as a versatile approach capable of independently eliminating primary tumors or lymphatic metastases in superficial tissues. While NIR light possess superior penetration depth compared to UV and visible light, it still faces limitations in tissue penetration. Despite its ability to reach approximately 3 centimetres deep into tissues, there remains a constraint on the depth to which NIR light can effectively penetrate. Overcoming the limited tissue penetration of near-infrared (NIR) light in deep tissues presents a significant hurdle for achieving the complete eradication of metastatic cancer cells or metastatic nodules in distant organs through PTT alone. To enhance the effectiveness in combating cancer metastasis, it is imperative to integrate photothermal therapy (PTT) with existing therapeutic modalities.

In recent years, photothermal nanomaterials in the NIR-I (750-900 nm) have had considerable interest due to the high biological penetrability of NIR light. However, the penetration depth of this NIR region is insufficient for solid tumors due to high scattering. PTT using NIR-II materials has drastically developed as a novel cancer therapy. The wavelength spectrum in the NIR-II region (1000-1700 nm) has a higher PCE potential and radiation limit and facilitates extensive tissue infiltration. It also has greater tissue sensitivity than the light in the NIR-I region. The potential applications and development rate of materials in the NIR-II range are expected to expand significantly, offering promising advancements for photothermal treatment, albeit with a need for further material improvements.

The reasons behind the limited adoption of this approach (PTT) are not fully understood, but possible factors include inconsistencies in clinical outcomes, a perceived lack of superior effectiveness compared to other local ablation methods or surgery, and the inherent complexity of the treatment, which involves both a drug and a device. In contrast, there have been successful developments in medical devices for cancer photothermal therapy (PTT) that do not necessitate the use of contrast agents. While contrast-enhanced PTT is a burgeoning area of research, its potential clinical utility is yet to be firmly established. Ongoing clinical trials, such as the one investigating gold nanoshells for prostate cancer ablation (NCT04240639), indicate sustained interest and investment in exploring this avenue further.

Eliminating the necessity for a photothermal contrast agent (PTT) presents a notable advantage in streamlining regulatory strategies and diminishing development costs. Currently, there exists a divergence between the emphases of preclinical and clinical PTT studies. Preclinical investigations predominantly focus on characterizing novel photothermal contrast agents, whereas clinical studies primarily concentrate on devising integrated laser ablation systems that do not depend on exogenous contrast. This incongruity may arise from the demonstrable feasibility of contrast-dependent PTT tumor ablation in preclinical models, enabling swift, reproducible, and resource-efficient testing of diverse compounds and materials. However, it is noteworthy that while phase I trials involving gold nanoshells demonstrate the potential of PTT agents, their clinical development lags considerably behind that of photodynamic therapy (PDT) and contrast-free laser thermal therapy.

Utilizing laser devices alone offers a viable approach for administering PTT in cancer treatment. For instance, Nd:YAG and other lasers can be employed for endoscopic irradiation of obstructing endobronchial cancers, facilitating their ablation through photocoagulation^98^.

In conclusion, PTT will fulfill the need for antitumor treatment, and safety concerns will be monitored and eliminated in the coming years of research. However, the upcoming research mainly focuses on the combinatorial effect of PTT with other cancer therapies as they enhance clinical efficiency and destroy the tumor cells within the laser irradiation region to achieve excellent therapeutic results than monotherapy (PTT alone). Moreover, combined therapies demonstrate heightened therapeutic efficacy; however, a deeper exploration is essential to understand the mechanisms underlying synergistic effects and to optimize them further. Use of LED lights instead of laser will be promoted due to their affordability, and mild hypothermia to reduce the side effects associated with PTT.

## Figures and Tables

**Table 1:** The list of bio-nanomaterials used in PTT

Photothermal Agents	Formulation	Type of cancer	Therapeutic marks	Reference
1. Carbon-based NMs				
a) Carbon Nanotubes (CNTs)				
i) SWNTs	Se@CNTs	TNBC	Generation of ROS, which induced apoptosis of tumor cells	23
	ADP@SWNT/TNF	Any type	pDNA transfection capacity, good water dispersion stability	24
ii) MWNTs	CMWNTs-PEG	Lung cancer	Strong tumor targeting capacity, potent photo-thermal therapeutic efficacy	25
	O-CNT-PEG	Heart cancer	Lower cytotoxicity and reduced tumor size after PTT	21
b) Carbon dots				
	LRCDs	Breast cancer	Improved bioavailability and therapeutic properties	26
c) Graphene				
i) Graphene Nanosheet	rGO/MSN/PDA	Hepatocellular carcinoma	Enhanced release of the drug in the tumor site	27
ii) Graphene oxide	NCGO@DOX-FA NMs	Breast cancer	Prominent target specificity, good PCE, and photostability	28
	Go/BaHoF_5_/PEG	Cervical Carcinoma cancer	Can penetrate lesions through the EPR effect	29
iii) Reduced graphene oxide	TMX-Nano-rGO	Breast cancer	Good tumoricidal activity	30
	Plant extract-based rGO	Oesophageal adenocarcinoma cell lines (OE-19)	Successful destruction of tumour cells	31
iv) Graphene Quantum Dots	9T-GQDs	Breast cancer	High photothermal property	32
2. Small Molecular Organic Dyes				
a) Indocyanine Green				
	mPEG-luteolin-BTZ@ICG	Colorectal cancer	Destroy tumor cells	33
b) Cypate				
	SiO_2_-NH_2_@ICGNPs	HepG2 tumour cells	Significant killing of tumour cells	34
c) IR-780				
	Cyp-PMMA-Fe@MSCs	Lung cancer	PTT-enhanced radiotherapy	35
	Liplmage ^TM^815	HepG2 tumour cells	For deep tissue imaging	2
	IR-780/CFAc (PCIR) NPs	B 16 cell lines	High selectivity towards malignant cells	36
3. Proteins/ Peptides based NMs	PTX-ICG-HAS NPs	4T1 subcutaneous tumor	No toxicity or regrowth of tumors	37
4. Polymer-based NMs				
a) Conducting polymers				
i) PANI	GG@PANI(Fe)-borax)	Skin cancer	Controlled release of the drugs	38
	Au nanostar@polyaniline	4T1 cells	Tremendous PCE	39
ii) Polypyrrole	PPR_ILK_	Papillary thyroid cancer	Lymphatic metastasis inhibition	40
b) Melanin-like polymers				
	Melanin PEGylated Nano liposomes	Skin cancer	Good biocompatibility	41
5.Microspheres of MXene	Ti3C_2_Tx MXenes	Breast cancer	Downregulated tumor progression	4

**Table 2:** The list of Graphene oxide nanoparticles used in PTT

S. No.	Sample	Therapeutic marks	References
1.	NGGO-FA	Excellent application prospects for tumor therapy	28
2.	GO/BaHoF_5_/PEG	Good PTT effect on cancer	29
3.	PEG-CMC/HAP/GO	Effectively inhibit tumor cell proliferation	52
4.	PNIPAM/GO	Biocompatible with less toxicity against tumor	53

**Table 3:** The list of reduced graphene oxide nanomaterials used in PTT

S. No.	Sample	Therapeutic marks	References
1.	Nano-rGO	Kill the tumor cells	30
2.	RNGO	Kill OE-19 cell	31
3.	rGO@msilica	Powerful platform for cancer therapy	55
4.	P-DOPA-rGO	For Application in breast cancer, PTT	57
5.	IR780-rGO/HA	For synergistic CT/PTT/PDT therapy	56
